# Clinical utility of cell-free urine miR-93-5p, miR-191-5p, miR-31-5p for invasive urothelial carcinoma detection and immune signature-based subtyping

**DOI:** 10.1186/s12894-026-02047-y

**Published:** 2026-01-15

**Authors:** Sami Berk Özden, Aysel Kalaycı, İclal Gürses, Filiz Özdemir, İpek Sertbudak, Furkan Kuzucu, Çetin Demirdağ

**Affiliations:** 1 Department of Urology, Osmaniye State Hospital, Osmaniye, Turkey; 2 Department of Medical Genetics, Cerrahpasa Medical Faculty, Istanbul, Turkey; 3 Department of Pathology, Cerrahpasa Medical Faculty, Istanbul, Turkey; 4 Department of Urology, Cerrahpasa Medical Faculty, Istanbul, Turkey

**Keywords:** Cell-free miRNA, Bladder cancer, Luminal, Basal, Non-invasive biomarker

## Abstract

**Purpose:**

This study aimed to explore the potential value of urinary cell-free microRNA (miR)-93-5p, miR-191-5p, and miR-31-5p levels in the non-invasive prediction of basal-like and luminal-like invasive urothelial carcinoma immune signature phenotypes according to the molecular classification of bladder cancer, by comparing their expression in patients and healthy controls.

**Methods:**

The study included morning urine samples from 49 bladder cancer patients and 43 controls. A quantitative image-based immunohistochemical analysis classified tumor cases into basal-like and luminal-like subtypes. Quantitative real-time PCR (qRT-PCR) was used to measure cell-free miR-93-5p, miR-191-5p, and miR-31-5p expression levels in urine.

**Results:**

MiR-191-5p was significantly downregulated in bladder cancer patients compared to healthy controls (*p* < 0.001), with a 24-fold decrease. Notably, miR-191-5p levels were markedly lower in the luminal-like subtype relative to the control group (*p* < 0.001). In contrast, miR-93-5p was significantly upregulated in the basal-like subtype, showing a 4.15-fold increase compared to controls (*p* < 0.001). Elevated levels of miR-93-5p were also observed in high-grade tumors (3.4-fold, *p* = 0.004), in tumors exhibiting necrosis (3.4-fold, *p* < 0.001), and in the presence of carcinoma in situ (CIS) (2.6-fold, *p* = 0.02). Furthermore, miR-93-5p levels showed a positive correlation with tumor size (*r* = 0.41, *p* < 0.001). In this exploratory cohort, miR-93-5p also demonstrated strong discriminatory performance in identifying CK5/6-positive tumors, with a receiver operating characteristic (ROC) curve cut-off value of 1.57 for 2^^ΔΔ^CT value.

**Conclusions:**

In this single-center, exploratory study, urinary cell-free miR-93-5p and miR-191-5p showed potential utility as non-invasive biomarkers for rapid molecular subtype identification in bladder cancer. miR-93-5p was associated with basal-like (aggressive) tumor features, while miR-191-5p was inversely associated with invasive urothelial carcinoma. miR-31-5p appears to serve as a complementary marker, especially in cases of CIS.

**Supplementary Information:**

The online version contains supplementary material available at 10.1186/s12894-026-02047-y.

## Introduction

Urothelial carcinoma (UC) is a significant global health concern, ranking as the ninth most common malignancy worldwide and one of the leading causes of mortality among urological cancers [1]. Representing the predominant histological subtype of bladder cancer, UC is characterized by marked biological heterogeneity and clinical variability [2]. This diversity poses substantial challenges in the areas of diagnosis, prognostication, and treatment planning, often complicating therapeutic decision-making and follow-up strategies [2–4].

The advent of next-generation sequencing (NGS) technologies has revolutionized the molecular understanding of invasive UC. Early transcriptomic analyses classified invasive UC into two major subtypes: luminal and basal, each with distinct gene expression profiles and clinical behavior. Luminal tumors characteristically express markers associated with differentiated urothelium, while basal tumors are associated with higher levels of aggressiveness and a poorer prognosis [3–6].

Molecular classification frameworks, such as those developed by The Cancer Genome Atlas (TCGA), MD Anderson Cancer Center, and Lund University, have provided more profound insights into UC subtypes using comprehensive transcriptomic and multi-omics data [4–8]. Despite discrepancies in nomenclature and subgrouping, these models have been consistently shown to identify luminal tumors by the expression of markers such as *KRT20*,* PPARG*,* FOXA1*,* GATA3*,* UPK1A*,* UPK2*, and *FGFR3*. Conversely, basal tumors are characterized by the expression of KRT5, KRT6, KRT14, and TP63, often accompanied by mutations in TP53 and RB. Importantly, immunohistochemical (IHC) evaluation of markers such as CK5, CK6, CD44, p63, RB, p53 (basal), and FGFR3, FOXA1, GATA3, CK20, CK14 (luminal) offers a practical surrogate for transcriptomic profiling in clinical settings [8].

MicroRNAs (miRNAs) have emerged as crucial regulators of gene expression and key contributors to cancer pathogenesis. These small, non-coding RNA molecules function in post-transcriptional regulation and exhibit both oncogenic and tumor-suppressive roles depending on the target transcript [9, 10]. Among various biological fluids, urine is particularly well-suited for miRNA-based biomarker development due to its non-invasive accessibility and rich content of tumor-derived nucleic acids [11]. Recent prospective studies and meta-analyses have underscored the utility of urine-based miRNA panels in the early diagnosis of bladder cancer [12–17]. Beyond their diagnostic potential, studies have shown that some microRNAs are therapeutically important [18–20], and seminal cohort studies have also validated urinary miRNAs as robust biomarkers for invasive UC. Cabrera et al., in a cohort of 120 patients, identified miR-145, miR-143, miR-125b, and miR-99a as significantly overexpressed in invasive UC, alongside oncogenic miR-182 and miR-96 [21]. Similarly, Juracek et al., using a comprehensive miRNA panel in a larger cohort of 304 patients, demonstrated elevated urinary levels of miR-31-5p, miR-93-5p, and miR-191-5p in invasive UC patients compared to controls [22]. These three miRNAs play roles in key pathways that directly contribute to tumor aggressiveness. miR-93-5p is a documented nucleotide that promotes aggressive phenotypes by targeting tumor suppressors, such as PTEN, which can be dysregulated in basal-subtype cancers [23]. miR-191-5p exerts a pro-oncogenic influence by disrupting apoptotic signals, including those mediated by p53 and SOX4, thereby promoting more aggressive disease [24]. Furthermore, miR-31-5p has a direct role in the regulation of KRT6, which is a basal urothelial marker [25]. Their differential expression in urine thus holds promise as a liquid biopsy reflection of the tumor’s core molecular subtype. Building on evidence for the biomarker potential of these specific miRNAs and their established roles in tumorigenesis, the purpose of this study is to test the novel hypothesis that urinary levels of miR-31-5p, miR-93-5p, and miR-191-5p are differentially expressed between basal-like and luminal-like subtypes of invasive UC.

## Methods

### Patient enrollment and sample collection

This prospective cohort study was conducted at the Cerrahpasa Medical Faculty Department of Urology clinic between January 2022 and August 2023 and evaluated the utility of urinary cell-free miR-31-5p, miR-93-5p, and miR-191-5p as biomarkers for predicting subtype-related immune staining patterns in invasive UC.

The patient group comprised adults (≥ 18 years) presenting with clinical or radiological suspicion of bladder tumor scheduled for transurethral resection of bladder tumor (TURBT), meeting specific inclusion criteria: histologically confirmed invasive UC (stage ≥ T1) after initial TURBT, no prior urinary tract malignancy, absence of significant chronic urinary tract diseases, fitness for general anesthesia, and provision of written informed consent. A control group of healthy adults (≥ 18 years), matched for age and sex, was recruited from the same clinic with inclusion criteria of no history of bladder tumor or any other malignancy, no significant chronic urinary tract disease, no use of medications with active urinary metabolites, and provision of consent. Exclusion criteria applied to both groups included active urinary tract infection, chronic indwelling catheterization, urolithiasis within the past 5 years, prior urothelial malignancy, active inflammatory disease, history of urinary tract surgery, or poor general medical condition contraindicating procedures.

**Fig. 1:**
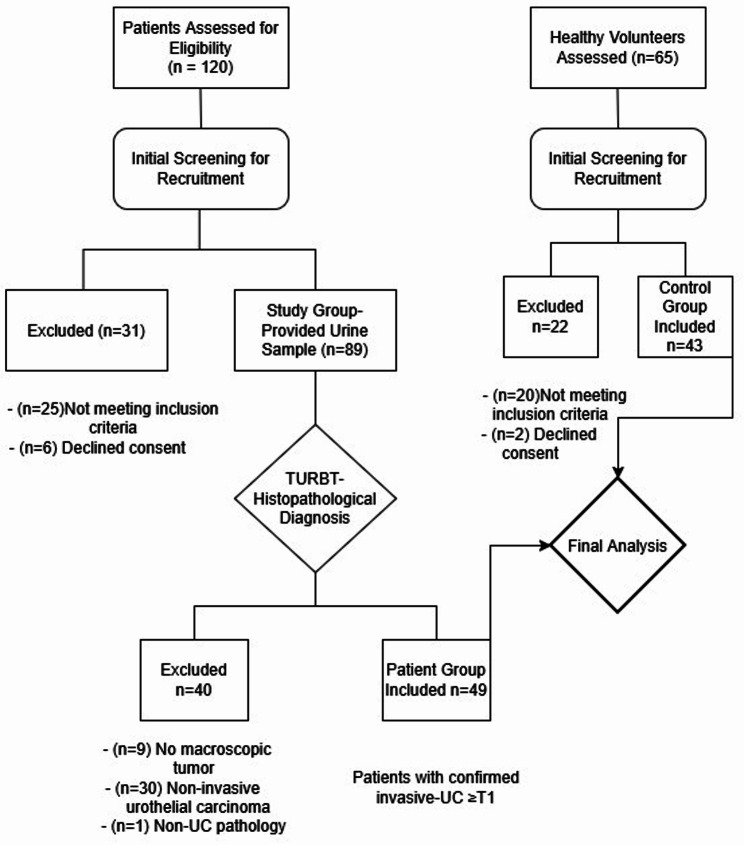
Patient flowchart

Prior to cystoscopy or any instrumentation, patients in the study group provided a first-void morning urine sample. These samples were immediately placed under appropriate preservation conditions and transported to the Department of Medical Genetics. The samples to be studied were immediately centrifuged at 3000 g for 20 min at 4 °C to remove cell debris, and the supernatant fluids were then collected and stored at − 80 °C until RNA isolation. Patients subsequently underwent cystoscopy; upon visual tumor confirmation, the pre-collected urine samples were preserved to undergo RNA extraction, miRNA-specific reverse transcription (cDNA synthesis), and qRT-PCR analysis. All patients then underwent standard TURBT, with resected tissue submitted for histopathological diagnosis; only patients with confirmed invasive UC (≥ T1) were included in the final analysis. Of the eighty patients with bladder tumors in our data, forty-nine met the inclusion criteria and were included in the study. In the control group, forty-three individuals met the study’s inclusion criteria Fig. 1.

### RNA isolation

Urine samples (300 mL) from patients collected into RNAase negative Nortgen Biotek Urine Preservation tubes with suspected primary bladder tumors were centrifuged at 300×g for 5–10 min. The pellet was removed, and the supernatant was isolated and recentrifuged (300×g, 5 min). Urine supernatant was collected, and total RNA was isolated using the miRNeasy Mini Kit (Qiagen, 217184) per the manufacturer’s protocol. Samples were processed immediately or stored at − 80 °C (≤ 3 days).

### cDNA synthesis

RNA (5 ng/µL) was reverse-transcribed using the miRCURY LNA RT Kit (Qiagen, 339340). Samples were split into four reactions, each with 2 µL of specific LNA primers for hsa-miR-93-5p (GenGlobe ID: YP00204715), hsa-miR-31-5p (GenGlobe ID: YP00204236), hsa-miR-191-5p (GenGlobe ID: YP00204306), or U6 snRNA (GenGlobe ID: YP02119464). Cycling periods consisted of: 42 °C (60 min) for RT activation, 95 °C (5 min) for denaturation, and 4 °C (10 min) for cooling.

### Quantitative real-time PCR

qRT-PCR was performed using the miRCURY LNA SYBR Green PCR Kit (Qiagen) on a 7900 HT system (Applied Biosystems). cDNA was diluted 1:60 in nuclease-free water. Reactions contained: SYBR Green Master Mix (5 µL), ROX Reference Dye (0.5 µL), and LNA primer (1 µL). Cycling was performed after initial UNG activation at 50 °C for 2 min, as follows: 95 °C (15 s.) for initial denaturation; 40 cycles of 95 °C (10 s.) for amplification and 60 °C (60 s.) for annealing.

Threshold cycle (Ct) values were normalized to U6 snRNA with the formula ΔCt (Ct microRNA- Ct U6 snRNA). Fold changes in microRNA expression were computed using the delta delta Ct method, implemented through REST 2009 R software (v4.0).

### Quantitative- image-based immunohistochemical analysis

IHC staining was performed on formalin-fixed paraffin-embedded tumor sections using protocols for six molecular subtype markers: p63 (clone 4A4, 1:1000 dilution; BioCare Medical), CK5/6 (clone D5/16B4, 1:50 dilution; Dako), p53 (clone DO-7, 1:500 dilution; Dako), RB (clone sc-73598, 1:500 dilution; Santa Cruz Biotechnology), GATA3 (clone HG3-31, 1:100 dilution; Santa Cruz Biotechnology), CK20 (clone Ks20.8, 1:400 dilution; Dako).

Antigen retrieval was performed using citrate buffer (pH 6.0) for p63, CK20, and p53; EDTA (pH 9.0) for CK5/6; Tris-EDTA (pH 9.0) for GATA3; and EDTA (pH 8.0) for RB. Stained slides were digitized using a PANNORAMIC 250 FLASH III slide scanner. The digitized images were analyzed using ImageJ software to calculate the labeling index, defined as the percentage of stained area. The results were validated by two pathologists.

The total area of immunostaining was quantified using the formula(labeling-index):

$$Percentage\:Positive\:Area\:=\:(Area\:of\:positive\:staining/Total\:tumor\:area)\times100$$ 

**Fig. 2:**
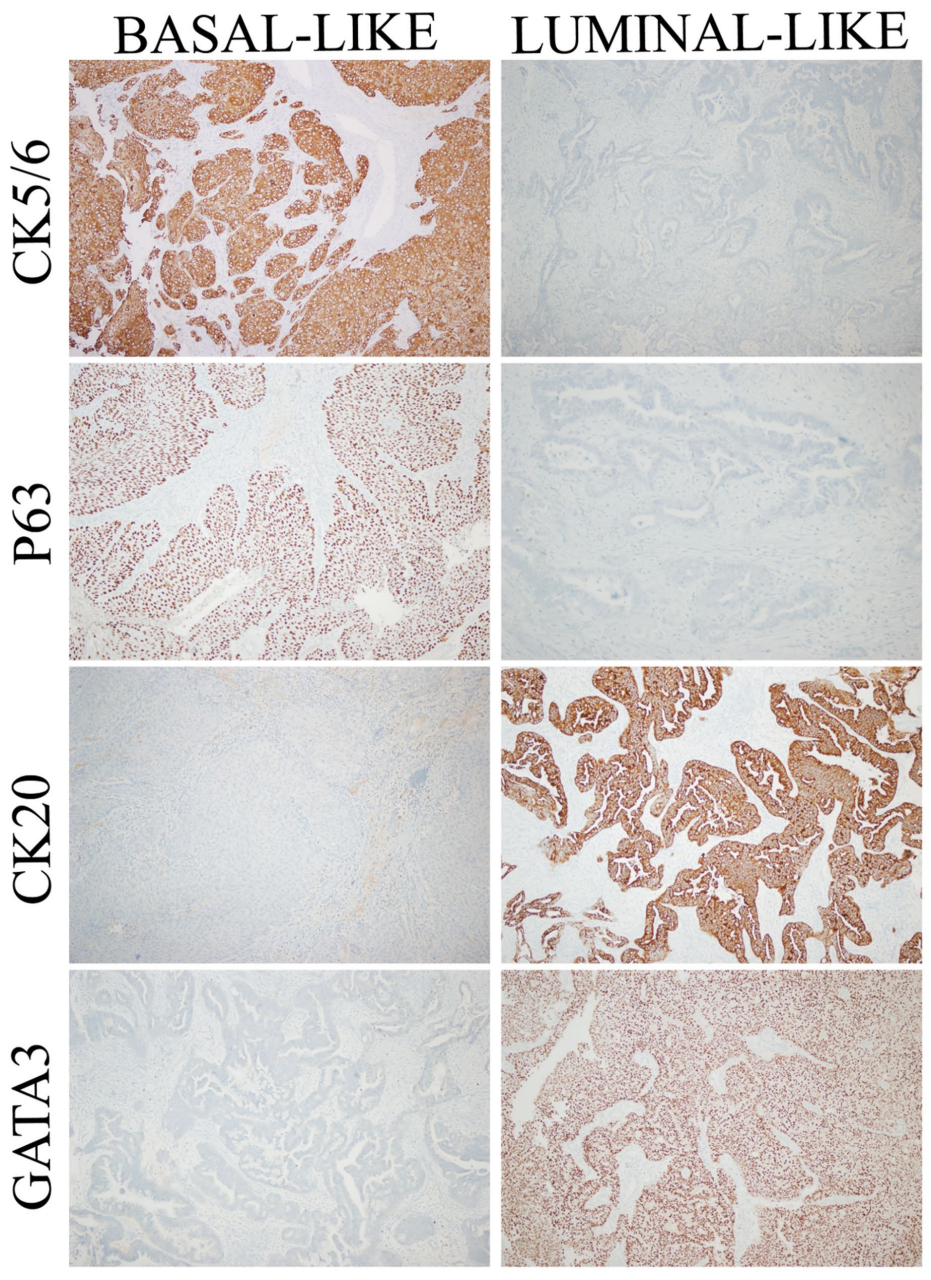
Representative immunohistochemical expression patterns of signature luminal and basal markers in luminal-like and basal-like urothelial carcinoma subtypes (images shown at x100 magnification)

**Fig. 3:**
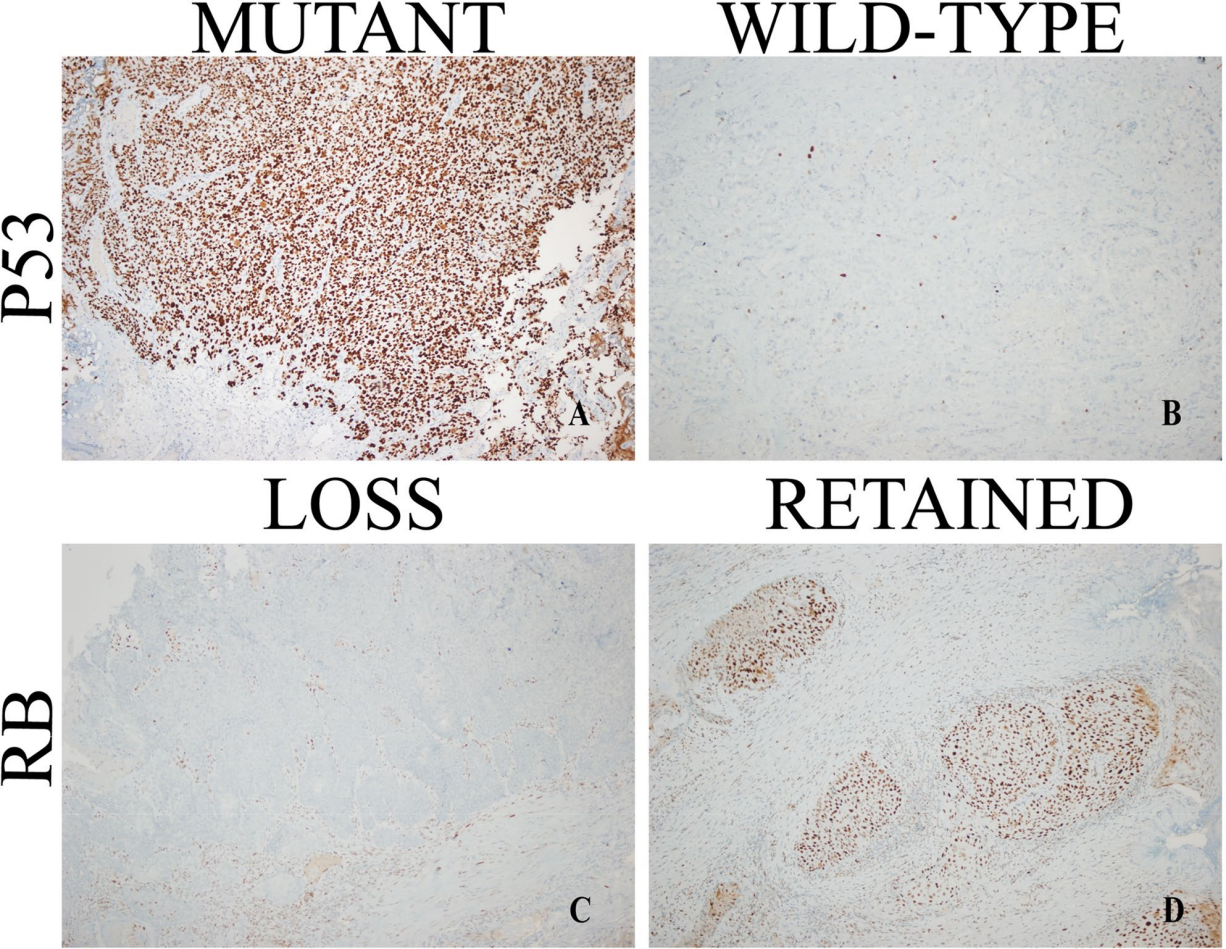
Representative immunohistochemical expression patterns: **A** p53 mutant-type showing strong diffuse nuclear positivity, **B** p53 wild-type with heterogeneous/negative staining, **C** RB loss demonstrating absent nuclear expression, and **D** RB retained with positive nuclear staining (all images at x100 magnification)

Tumors were sub-grouped as basal-like and luminal-like according to Dadhania et al.’s (2016) criteria [8]. CK5/6 or p63 expression was considered positive if ≥ 10% of tumor cells demonstrated cytoplasmic/membranous staining, and negative if < 10%. Both GATA3 (nuclear) and CK20 (cytoplasmic) required concurrent positivity, defined as ≥ 30% stained tumor cells each. Expression below 30% for either marker was classified as negative. Additionally, p53 staining status was classified as positive with any perceptible nuclear staining (wild-type or mutant pattern) and negative if completely absent. RB mutation status was deemed positive if there was loss of nuclear staining by IHC; retained staining was deemed negative Figs. 2 and 3.

The samples were classified as basal-like if they stained positive for any of the following markers: CK5/6, p63, p53, or RB. Tumors were classified as luminal-like only if they showed dual positivity for the luminal markers GATA3 and CK20 and did not meet any basal-like criteria Figs. 2 and 3.

## Statistics

Statistical analyses were performed using SPSS version 25.0 (IBM Corp., NY, USA). Normally distributed continuous variables are presented as mean ± standard deviation (SD). Non-normally distributed continuous variables (assessed using the Shapiro-Wilk test) are reported as median and interquartile range (IQR). Additionally, 95% confidence intervals (CI) and the minimum and maximum values are provided. Group comparisons used Mann-Whitney U tests, while comparisons for categorical variables were performed using the Chi-square test. Correlations with clinicopathological variables employed Spearman’s rank correlation. Diagnostic performance was evaluated via ROC curve analysis, reporting area under the curve (AUC), sensitivity, specificity, and optimal cutoffs. Fold-changes were calculated using the 2^ΔΔCT method (REST 2009). Significance was defined as *p* < 0.05 (two-tailed).

## Results

### Demographics and clinicopathological results

Patient demographics showed a matched age distribution cohort (mean age 68.14 ± 12.07 years, range 30–89) compared to controls (64.69 ± 9.84 years, range 31–80), with male predominance in both groups (patients: 87.8% male; controls: 83.7% male), as detailed in Table 1. IHC analysis of tumor specimens classified 57.1% (28/49) as luminal-like and 42.9% (21/49) as basal-like subtypes, with full marker expression profiles provided in Table 2. The stages of the patients were: 85.7% (42/49) were T1, 12.2% (6/49) T2, and 2% (1/49) T4. Other clinicopathological characteristics are summarized in Table 3.

**Table 1:** Demographic and clinical characteristics of the patient and control groups

Demographic	Patient Group (*n* = 49)	Control Group (*n* = 43)	*P*-Value
Age (years)	68.14 ± 12.07 (30–89)	64.69 ± 9.84 (31–80)	*P* = 0.078
Gender			*p* = 0.579
Male	43 (87.8%)	36 (83.7%)	
Female	6 (12.2%)	7 (16.3%)	
Smoking History			*p* < 0.001
Smokers	40 (81.6%)	3 (7.0%)	
Non-smokers	9 (18.4%)	40 (93.0%)	
Pack-years	25.40 ± 21.38 (0-120)	0.27 ± 1.09 (0–5)	*p* < 0.001
BMI	26.40 ± 3.25 (19.9–34.0)	25.17 ± 2.85 (19.5–33.4)	*p* = 0.059

Data presented as mean ± standard deviation (range) for continuous variables and n (%) for categorical variables

**Table 2:** Immunohistochemical marker expression in bladder cancer patients (*n* = 49)

Marker	Category	*n* (%)	*P*-Value
CK5/6	Positive	20 (40.8%)	*P* = 0.19
	Negative	29 (59.2%)	
GATA3	Positive	47 (95.9%)	*p* < 0.001
	Negative	2 (4.1%)	
p63	Positive	29 (59.2%)	*P* = 0.19
	Negative	20 (40.8%)	
CK20	Positive	41 (83.7%)	*p* < 0.001
	Negative	8 (16.3%)	
RB	Loss (Positive)	14 (28.6%)	*P* = 0.002
	Retained (Negative)	35 (71.4%)	
p53	Mutant Pattern	21 (42.9%)	*P* = 0.31
	Wild-type Pattern	28 (57.1%)	

Data are expressed as n (%). P-values correspond to Chi-square goodness-of-fit tests for the overall cohort

**Table 3:** Clinicopathological features of bladder cancer patients (*n* = 49)

Characteristic	Category	*n* (%)	*P*-Value
Initial T Stage	T1	42 (85.7%)	*p* < 0.001
	T2	6 (12.2%)	
	T4	1 (2.0%)	
Tumor Multiplicity	Solitary	22 (44.9%)	*p* = 0.47
	Multiple	27 (55.1%)	
Tumor Grade	High	40 (81.6%)	*p* < 0.001
	Low	9 (18.4%)	
Macroscopic Hematuria	Present	12 (24.5%)	*p* < 0.001
	Absent	37 (75.5%)	
Necrosis	Present	14 (28.6%)	*p* = 0.002
	Absent	35 (71.4%)	
CIS	Present	16 (32.7%)	*p* = 0.01
	Absent	33 (67.3%)	
LVI	Present	9 (18.4%)	*p* < 0.001
	Absent	40 (81.6%)	
Molecular Subtype	Luminal-like	28 (57.1%)	*p* = 0.317
	Basal-like	21 (42.9%)	

Data are expressed as frequencies with percentages in parentheses [n (%)]

*Abbreviations*: *CIS* Carcinoma in situ, *LVI* Lymphovascular invasion, *T Stage* Tumor stage

### MiRNA expression levels

**Fig. 4:**
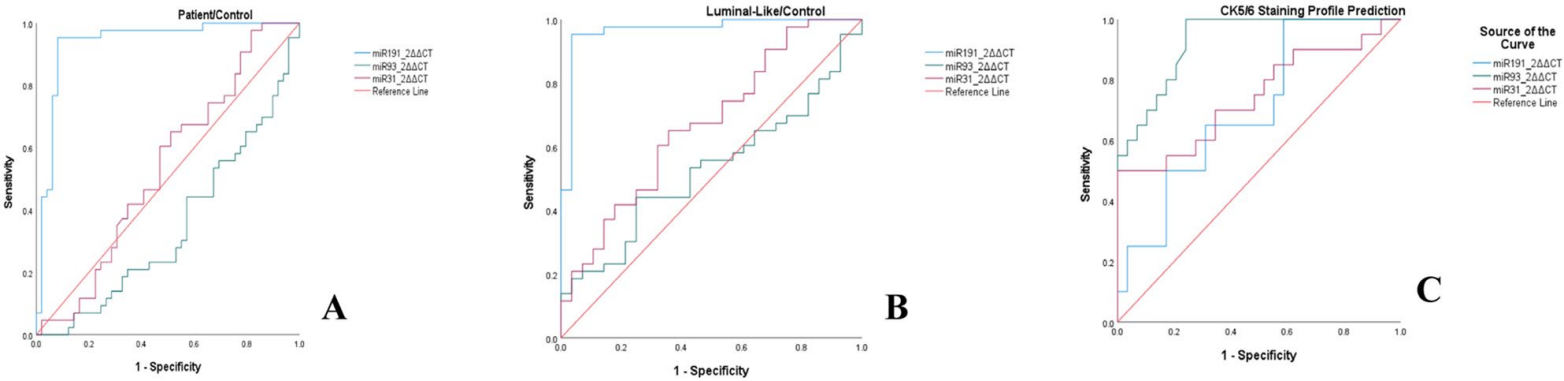
Receiver operating characteristic (ROC) curve analyses. **A** miRNA distinguishing patients from controls. **B** miRNA distinguishing the luminal-like subtype from controls. **C** miRNA predicting the CK5/6 staining profile

MiR-191-5p demonstrated significant downregulation in all invasive UC patients versus controls (24-fold decrease, median 0.0397 [0.543, 0.042–0.165] vs. 0.97 [1.40, 1.096–2.086], IQR, 95% CI *p* < 0.001). ROC curve yielded an AUC = 0.937, 95% CI: 0.882–0.992. At an optimal cutoff of < 0.258 2^^ΔΔ^CT, sensitivity reached 95.3%, specificity 91.8%, yielding a positive likelihood ratio of 11.62 Fig. 4.

Concomitantly, miR-191-5p showed 28.37-fold reduction in the luminal-like group compared to controls (median 0.034 [0.035, 0.005–0.147] vs. 0.970 [1.40, 1.096–2.086], IQR, 95% CI *p* < 0.001). ROC curve yielded an AUC = 0.967, 95% CI: 0.923–1. 000. At an optimal cutoff of < 0.206 (2^^ΔΔ^CT), sensitivity reached 95.3%, specificity 96.4%, yielding a positive likelihood ratio of 26.5 Fig. 4.

MiR-191-5p upregulation was found to be statistically significantly higher in patients with T-upstaging (1.73-fold, *p* = 0.03), N-upstaging (3.58-fold, *p* = 0.02), and M-upstaging (4.08-fold, *p* = 0.03) through a 1-year time frame Table 4.

**Table 4:** Comparison of MiRNAs with clinical and pathological features

	miR-93-5p	miR-191-5p	miR-31-5p
MOLECULAR SUBTYPES			
Basal-like vs. Luminal-like	**↑**4.5× (*p* < 0.001) 4.15 (1.85–111.61) 0.92(0.21–3.36)	**↑**2.1× (*p* = 0.007) 0.03 (0.01–4.48) 0.07 (0.02–0.19)	**↑**3.1× (*p* = 0.009) 1.77 (0.07–74.77) 0.58 (0.05–2.80)
TUMOR GRADE			
High vs. Low	**↑**3.4× (*p* = 0.004) 2.40 (0.24–111.61) 0.71 (0.21–2.68)	**↓**1.4× (*p* = 0.03) 0.14 (0.02–4.48) 0.10 (0.01–0.34)	NSD (*p* = 0.28) 0.84 (0.05–74.77) 0.56 (0.18–1.99)
TUMOUR CHARACTERISTICS			
Size	*r* = 0.41 (*p* < 0.001)	NSD (*p* = 0.73)	*r* = 0.37 (*p* = 0.01)
Multiplicity (Multiple vs. Solitary)	NSD (*p* = 0.81) 2.10 (0.24–111.61) 1.57 (0.21–8.12)	NSD (*p* = 0.31) 0.12 (0.02–4.48) 0.12 (0.01–1.02)	NSD (*p* = 0.78) 0.79 (0.07–74.77) 0.68 (0.05–8.78)
Macroscopic Hematuria (Present vs. Absent)	**↑**2.95× (*p* = 0.01) 3.34 (0.81–111.61) 1.13 (0.21–12.93)	NSD (*p* = 0.09) 0.24 (0.06–4.48) 0.12 (0.01–2.83)	**↑**2.06× (*p* = 0.05) 1.34 (0.10–74.77) 0.65 (0.05–8.78)
IHC PHENOTYPES			
CK5/6(Stained vs. Not Stained)	**↑**4.1× (*p* < 0.001) 3.81 (1.13–111.61) 0.93 (0.21–3.49)	**↑**1.9× (*p* < 0.001) 0.19 (0.08–4.48) 0.10 (0.01–2.83)	**↑**3.0× (*p* = 0.01) 1.67 (0.07–74.77) 0.56 (0.05–2.80)
P-53 Mutation (Mutant vs. Wild-Type)	**↑**3.5× (*p* < 0.001) 3.37 (0.41–111.61) 0.97 (0.21–6.24)	NSD (*p* = 0.08) 0.13 (0.02–4.48) 0.10 (0.01–2.83)	NSD (*p* = 0.08) 1.32 (0.09–74.77) 0.72 (0.05–6.89)
RB (Stain Loss vs. Retain)	**↑**2.3× (*p* = 0.03) 2.61 (0.56–27.14) 1.13 (0.21–111.61)	NSD (*p* = 0.34) 0.13 (0.06–2.05) 0.12 (0.01–4.48)	NSD (*p* = 0.41) 0.99 (0.07–9.09) 0.73 (0.05–74.77)
CK20(Stained vs. Not Stained)	**↓**3.6× (*p* = 0.01) 1.28 (0.21–27.14) 4.57 (1.92–111.61)	NSD (*p* = 0.27) 0.12 (0.01–2.83) 0.16 (0.08–4.48)	**↑**3.5× (*p* = 0.04) 0.73 (0.05–14.77) 2.55 (0.49–74.77)
GATA-3(Stained vs. Not Stained)	NSD (*p* = 0.08) 2.03 (0.21–111.61) 5.62 (5.00–6.24)	NSD (*p* = 0.42) 0.12 (0.01–4.48) 0.24 (0.13–0.34)	NSD (*p* = 0.06) 0.77 (0.05–74.77) 6.36 (3.93–8.78)
P63(Stained vs. Not Stained)	NSD (*p* = 0.17) 2.42 (0.21–111.61) 1.10 (0.43–6.24)	NSD (*p* = 0.24) 0.13 (0.01–4.48) 0.11 (0.03–2.83)	NSD (*p* = 0.79) 0.77 (0.07–74.77) 0.84 (0.05–8.78)
PATHOLOGIC FEATURES			
Necrosis(Present vs. Absent)	**↑**3.4× (*p* < 0.001) 3.82 (1.07–111.61) 1.11 (0.21–8.83)	**↑**2.7× (*p* < 0.001) 0.30 (0.08–4.48) 0.11 (0.01–2.83)	NSD (*p* = 0.39) 0.77 (0.10–74.77) 0.79 (0.05–14.77)
CIS(Present vs. Absent)	**↑**2.6× (*p* = 0.02) 2.84 (0.43–12.93) 1.10 (0.21–111.61)	NSD (*p* = 0.52) 0.14 (0.03–0.49) 0.12 (0.01–4.48)	**↑**2.77× (*p* = 0.03) 1.80 (0.05–8.78) 0.65 (0.07–74.77)
LVI(Present vs. Absent)	NSD (*p* = 0.19) 2.61 (0.56–6.24) 1.42 (0.21–111.61)	**↑**2.4× (*p* = 0.02) 0.29 (0.06–0.49) 0.12 (0.01–4.48)	NSD (*p* = 0.08) 1.77 (0.40–14.77) 0.75 (0.05–74.77)
PATIENT FACTORS			
Smoking status (Smoker vs. Non-smoker)	NSD (*p* = 0.17) 1.92 (0.24–111.61) 1.05 (0.03–27.14)	**↓ **7.1× (*p* < 0.001) 0.12 (0.02–5.29) 0.85 (0.01–7.12)	NSD (*p* = 0.82) 0.80 (0.07–74.77) 0.85 (0.05–26.99)
Pack-years	NSD (*p* = 0.24)	*r*=-0.47 (*p* < 0.001)	NSD (*p* = 0.74)
Charlson Comorbidity Score	NSD (*p* = 0.08)	*r*=-0.44 (*p* < 0.001)	NSD (*p* = 0.16)
PROGNOSIS			
T-upstaging (Yes vs. No)	NSD (*p* = 0.10) 2.67 (0.21–12.93) 1.11 (0.24–111.61)	**↑**1.73× (*p* = 0.03) 0.19 (0.01–1.02) 0.11 (0.02–4.48)	NSD (*p* = 0.95) 0.69 (0.07–14.77) 0.80 (0.05–74.77)
N-upstaging(Yes vs. No)	NSD (*p* = 0.18) 2.51 (0.56–27.14) 1.57 (0.21–111.61)	**↑**3.58× (*p* = 0.02) 0.43 (0.06–2.05) 0.12 (0.01–4.48)	NSD (*p* = 0.23) 2.56 (0.07–14.77) 0.77 (0.05–74.77)
M-upstaging(Yes vs. No)	NSD (*p* = 0.14) 3.37 (0.56–27.14) 1.75 (0.21–111.61)	**↑**4.08× (*p* = 0.03) 0.49 (0.06–2.05) 0.12 (0.01–4.48)	NSD (*p* = 0.30) 1.77 (0.07–9.09) 0.75 (0.05–74.77)

All comparisons used Mann-Whitney U tests (↑ and ↓ indicate increased or decreased miRNA expression (fold-change)); correlations report Spearman’s r.

*Abbreviations*: *CIS*  Carcinoma In Situ, *LVI*  Lymphovascular Invasion

*NSD* Non-Significant Difference (*p* > 0.05)

**Fig. 5:**
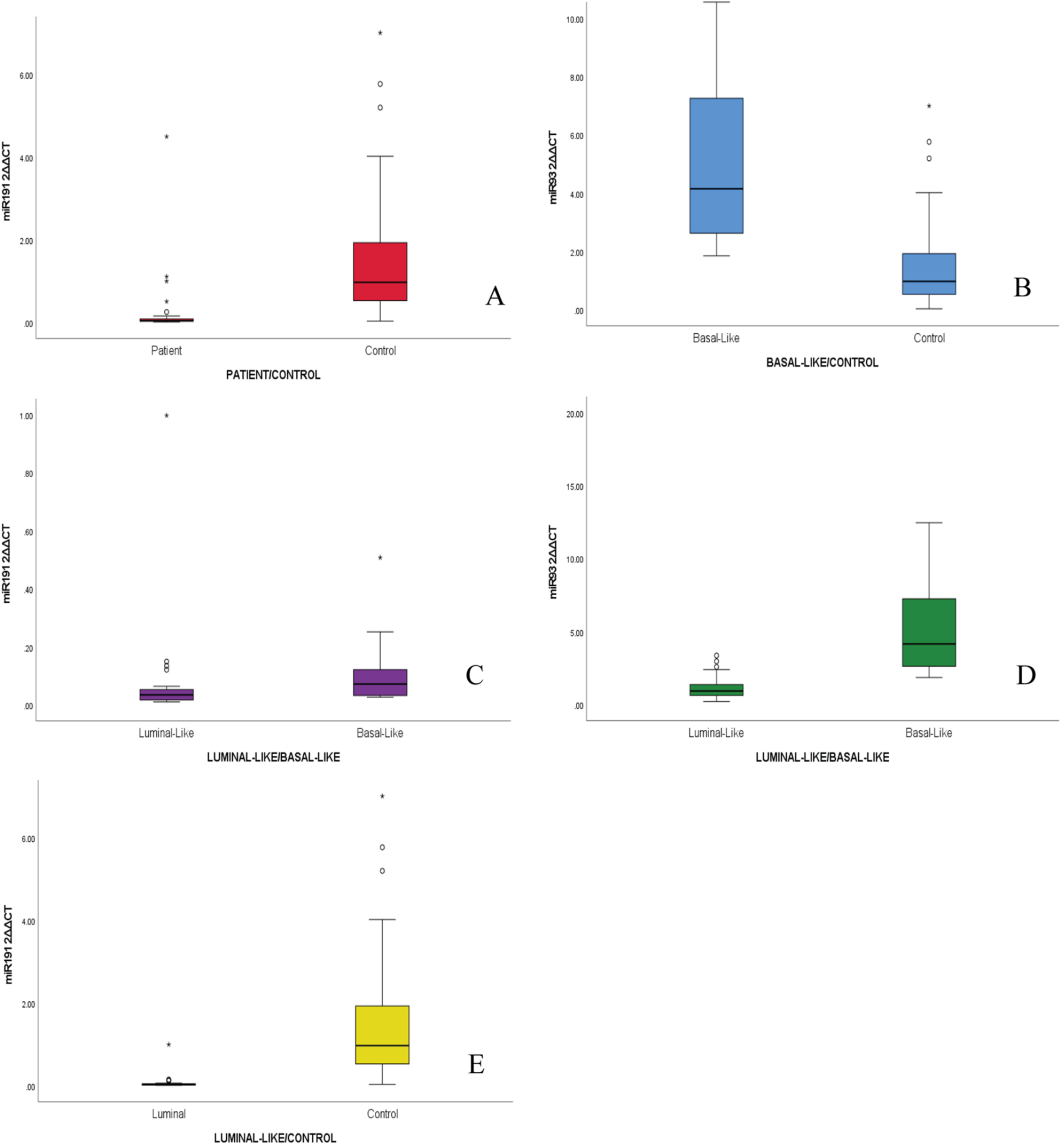
Boxplots comparing miRNA expression levels between patient groups and controls, stratified by characteristics. **A** miR-191-5p levels in bladder cancer patients versus the control group. **B** miR-93-5p levels in the basal-like subgroup versus controls. **C** miR-191-5p levels comparing basal-like and luminal-like subgroups. **D** miR-93-5p levels in the luminal-like subgroup versus controls. **E** miR-191-5p levels in the luminal-like subgroup versus controls

MiR-191-5p levels also expressed a 1.44-fold reduction in luminal-like group in comparison to basal-like subtypes (median 0.034 [0.035, 0.005–0.147] vs. 0.049 [0.085, 0.028–0.260], IQR, 95% CI, *p* = 0.007) Fig. 5.

MiR-93-5p was found to be 4.3-fold elevated in basal-like tumors versus control group (median 4.15 [4.97, -0.007, − 21.588] vs. 0.970 [1.4, 1.097–2.086], IQR, 95% CI *p* < 0.001) and 4.5-fold higher in basal-like tumors than luminal-like tumors (median 4.15 [4.97, -0.007 − 21.588] vs. 0.929 [0.846, 0.845–1.499], IQR, 95% CI *p* < 0.001) Fig. 5.

MiR-93-5p was again found to be elevated to 3.4-folds in high-grade tumours (median 2.40 vs. 0.71, *p* = 0.004), miR-191-5p on the other hand was 1.4-fold lower in low-grade tumours than the high-grade tumours (*p* = 0.03). miR-93-5p was deemed to increase with tumour size (*r* = 0.41, *p* < 0.001), presence of necrosis (3.4-fold, *p* < 0.001), and accompanying CIS (2.6-fold, *p* = 0.02), yet showed no difference relative to tumour count (solitary or multiple) or macroscopic hematuria status (present or absent). (*p* > 0.05) miR-191-5p was also slightly increased in the presence of lymphovascular invasion (LVI) (2.4-fold increase, *p* = 0.02) and necrosis (2.7-fold, *p* < 0.001) Table 4.

MiR-31-5p levels between the patient group and the control group were found to be statistically insignificant and lacked discriminatory power to determine patients with UC. (*p* > 0.05) However, among patients with invasive UC, expression levels were significantly higher in those with basal-like tumors compared to luminal-like tumors, showing a 3.1-fold increase (median 1.77[5.96, -0.35-14.22] vs. 0.57[0.85, 0.47–0.99], IQR, 95% CI *p* < 0.001). Elevated levels of miR-31-5p were significantly associated with the presence of CIS (2.7-fold increase, *p* = 0.03) and positively correlated with tumor size (*r* = 0.37, *p* < 0.05) Table 4.

**Fig. 6:**
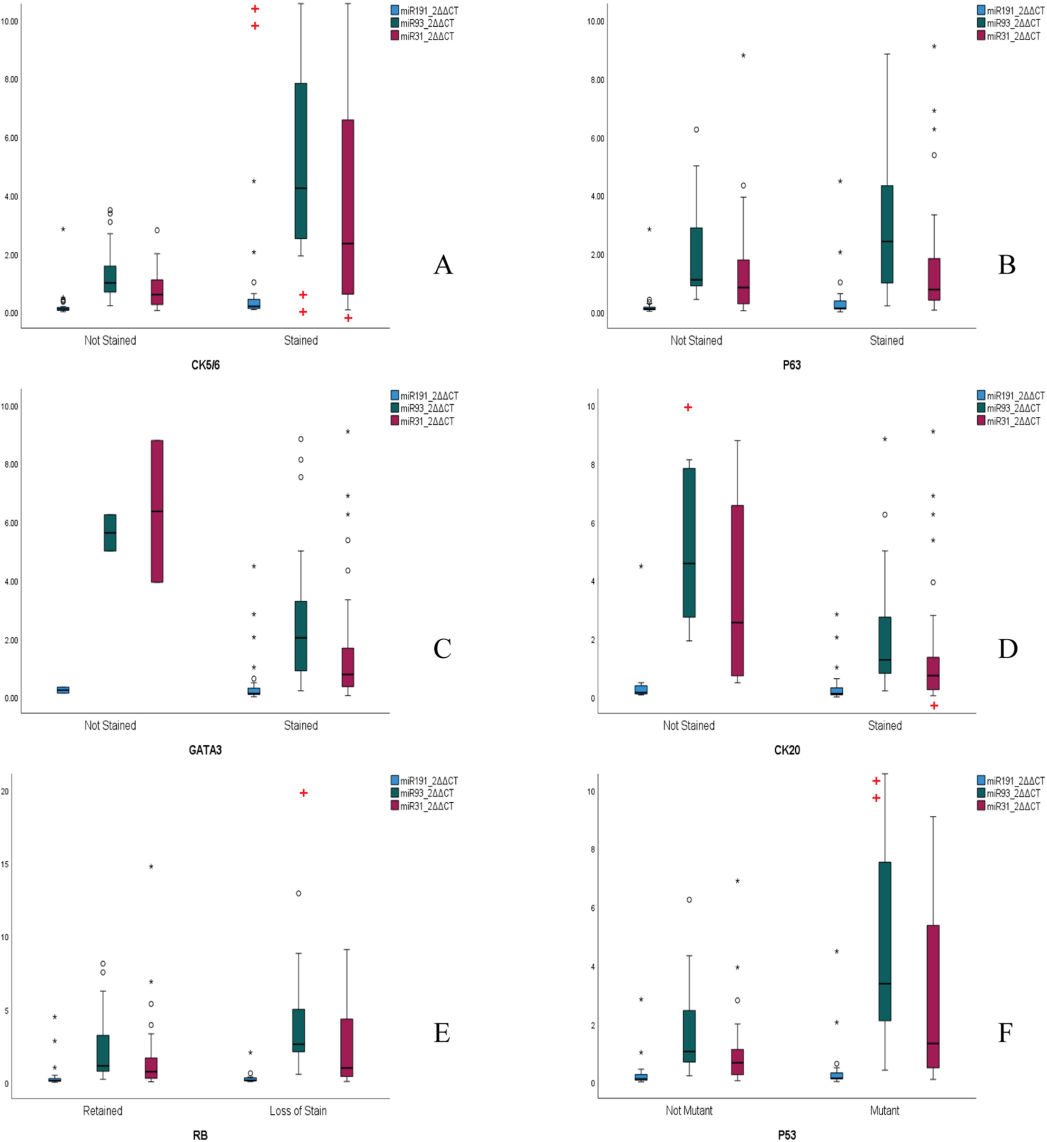
Boxplots comparing miRNA expressions according to quantitative immunohistochemistry staining patterns across the patients. **A** CK5/6 expression. **B** P63 expression. **C** GATA3 expression. **D** CK20 expression. **E** RB expression. **F** P63 expression. Statistically significant differences between compared groups, as assessed by the Mann-Whitney U test, are indicated directly on the figure with red cross symbols: +(p < 0.05); ++(p < 0.001)

Statistically significant differences between miRNA expressions for staining patterns were also observed. miR-93-5p expressions were found to be 4.1-fold higher in CK5/6-positive tumors (*p* < 0.001) with ROC analysis confirming remarkable discrimination (AUC = 0.928, 95%CI:0.82–0.98). At the optimal cutoff > 1.57 2^ΔΔCT, sensitivity reached 95.45% [95%CI:77.2–99.9%] and specificity 77.78% [95%CI:57.7–91.4%], yielding a positive likelihood ratio of 4.30 Fig. 4. Similarly, p53-mutant tumors exhibited 3.5-fold elevated miR-93-5p levels (median 3.37 vs. 0.97; *p* < 0.001), while urine samples of patients that had RB loss showed 2.3-fold upregulation (median 2.61 vs. 1.13; *p* = 0.03) Fig. 6.

Nevertheless, CK20-negative tumors showed 3.6-fold higher miR-93-5p expression (median 4.57 vs. 1.28; *p* = 0.01). In contrast to its low expression on urothelial tumors, miR-191-5p was 1.9-fold elevated in CK5/6 + tumors (median 0.19 vs. 0.10; *p* < 0.001) with fair diagnostic power (AUC = 0.774, 95%CI:0.63–0.88). At an optimal cutoff of 0.121 (2^ΔΔCT), sensitivity reached 75.0%, specificity 65.5%, yielding a positive likelihood ratio of 2.17 Fig. 4. miR-31-5p also showed 3.0-fold increase in CK5/6 + cases with marginal significance(*p* = 0.01). Neither miRNA correlated with GATA3 expression (miR-93-5p *p* = 0.08; miR-191-5p *p* = 0.42) or p63 staining profile (both *p* > 0.05) Fig. 6.

Clinically, there was no significant difference between smokers and non-smokers in the expression of miR-93-5p or miRNA-31-5p. However, a significant reduction was observed for miR-191-5p, with smoker patients showing a 7.1-fold lower expression compared to non-smokers. Furthermore, Charlson’s comorbidity score demonstrated no statistically significant variation between patients for the three levels of microRNA (*p* > 0.05). Table 4.

## Discussion

MicroRNAs (miRNAs) are small, non-protein-coding RNA molecules, typically 20–24 nucleotides in length. They regulate gene expression by promoting degradation or suppressing the translation of target messenger RNAs (mRNAs) and can function either as oncogenes or tumor suppressors. miRNAs can be isolated from various biological samples, including blood plasma, urine, stool, and biopsy specimens. They are generally stable and resistant to diverse storage conditions, and their alterations can be reliably detected in body fluids. Owing to these properties, miRNAs hold significant potential as molecular markers for diagnosis, prognosis, and clinical monitoring [26]. 

In this study, we provide exploratory evidence supporting the potential utility of cell-free miR-93-5p and miR-191-5p as non-invasive biomarkers for distinguishing between basal-like and luminal-like molecular subtypes in invasive UC, and demonstrate their association with key clinicopathological features of aggressiveness. Most existing studies on cell-free urinary miRNAs for bladder cancer have utilized limited patient cohorts and restricted miRNA panels [27–29]. Although studies with larger populations are now being conducted [21], a clear link between these miRNA profiles and the newly established molecular subtypes of bladder cancer has yet to be fully established. Largest cohort established in terms of cell-free miRNA Juracek et al. identified miR-31-5p, miR-93-5p, and miR-191-5p as being significantly elevated in urine from bladder cancer patients. They developed a diagnostic score, which was later refined into a two-miRNA model (miR-31-5p and miR-93-5p). However, despite incorporating clinicopathological variables such as tumor grade, the study population was not clinically homogeneous, as non-muscle invasive bladder cancer (NMIBC) and muscle-invasive bladder cancer (MIBC) were evaluated together, and macroscopic hematuria status was not considered, a factor that may influence the composition of the urinary supernatant. In addition, the study was primarily designed for cancer detection and did not integrate immune-related characteristics or contemporary molecular subtype frameworks, such as basal and luminal-like phenotypes [22]. Our study attempts to address this gap by focusing exclusively on invasive UC, accounting for macroscopic hematuria status, and incorporating quantitative immunohistochemical classification to link urinary miRNA profiles with immune-associated basal-like and luminal-like tumor subtypes.

Hsa-miR-93 is located at locus 22 on chromosome 7. After cleavage by the DICER enzyme, its 5’-3’ form is dominantly incorporated into the RNA-induced silencing complex (RISC) [30]. miR-93 potentially targets the mRNA of the E2F1 protein, the mRNA of the *p21* gene and its dependent TGFβ-mediated cell cycle suppression mechanism [31], and the mRNA of the *PTEN* gene and its role in mediating chemo-radio resistance [32], as well as the silencing of the *PEDF*-1 and *SERPIN* tumor suppressor genes, which are specifically effective in bladder cancer [33]. Nucleotide is also known to attenuate the immune reaction against tumor cells by targeting PD-L1 in breast cancer. Consistent with our study, miR-93-5p expression was significantly elevated in urine samples from patients with basal-like tumors, as defined by positive staining of immunohistochemical markers (CK5/6, p63, TP53, and RB loss). Our findings align with the established pro-oncogenic role of miR-93-5p across various cancers, including breast [34, 35], gastric [31], and esophageal [36] cancer. In breast cancer, miR-93-5p promotes tumorigenesis by epithelial-mesenchymal transition (EMT) [35] and inhibiting apoptosis, partly through its targeting of PD-L1 [34]. Furthermore, Yuan et al. demonstrated that miR-93-5p, which is known to suppress PTEN in breast cancer, 32 also potently downregulates PTEN in bladder cancer cells, thereby enhancing proliferation and invasion [37]. Our findings suggest that elevated miR-93-5p levels are significantly associated with clinicopathological features linked to aggressive disease, including larger tumor size (*r* = 0.41, *p* < 0.05), presence of tumor necrosis (*p* < 0.05), presence of P53 mutation (*p* = 0.0002), and RB1 loss (*p* = 0.03). The association of miR-93-5p with features of tumor aggressiveness, including loss and mutation of tumor suppressors (P53, RB1), is consistent with its documented pro-oncogenic role, which involves the progression of aggressive phenotypes and disruption of cell-cycle checkpoints [31, 33, 35, 37]. Despite its association with aggressive traits, levels of miR-93-5p were not affected by the presence of macroscopic hematuria or tumor multiplicity at initial presentation, supporting its candidacy as a potentially useful hematuria-resilient marker of invasive UC within basal-like features (*p* < 0.05). The clinical relevance of hematuria-independent urinary cell-free miRNA biomarkers has been previously explored, most notably by Piao et al., who demonstrated that the urinary miR-6124/miR-4511 expression ratio could discriminate bladder cancer from non-malignant hematuria; however, their analysis evaluated 27 miRNAs in a limited cohort of 35 patients comprising both NMIBC and MIBC. In contrast, our study focuses exclusively on invasive urothelial carcinoma and analyzes a larger, homogeneous cohort of 49 patients, with a more disease-specific assessment of hematuria-resilient miRNA [38]. 

Hsa-miR-191 is a miRNA with dual oncogenic/tumor-suppressive functions located at locus 21 on chromosome 3. In its stem-loop form, after being processed by the DICER enzyme, both its (3’-5’) form and its (5’-3’) form become attached to the RISC complex [30]. It has been found to suppress angiogenesis by activating the NF-κB pathway [39], alongside EMT, due to inhibition of oncogenic transcripts such as CDK9 and NOTCH2 [40]. Contrasting its anti-tumor properties, upregulation of this factor was found to cause tumor progression by promoting prostate cancer through suppressing TIMP3 and increasing metalloproteinase activity [41]. It was also found to mediate anti-apoptotic effects by suppressing p53 and SOX4 [24], and enhancing cell division through inhibiting DICER1, an essential component of canonical miRNA processing in breast cancer [42]. Our findings regarding the miR-191-5p in this study somewhat contradicted Juracek et al.‘s largest cohort [22]. Our study has shown that miR-191-5p levels were reduced in invasive UC patients, but specifically in luminal-like tumors (positive for GATA3/CK20), which may be due to its anti-tumor activity. Nonetheless, certain studies have reported miR-191-5p as a tumor-suppressor nucleotide in breast and prostate cancer, which aligns with the findings of our study, rendering miR-191-5p a negative marker for invasive UC and, more specifically, for carcinoma with luminal–immune signature staining [43, 44]. In parallel, large-scale exploratory efforts, as reported in a correspondence by Oto et al., identified miR-191-5p as part of a multi-miRNA urinary profile capable of identifying UC, while not specifically addressing subtype-associated immune features [45]. Moreover, miR-191-5p was observed to have potential prognostic relevance, as higher miR-191-5p levels were associated with an increased incidence of short-term tumor, node, metastasis (TNM) upstaging. This association aligns with the established pro-oncogenic role of miR-191-5p in bladder cancer, as reported in the literature; however, it somewhat contradicts our previous findings, which primarily identified nucleotides within its tumor-suppressor traits. This paradoxical discrepancy may be due to its dual oncogenic/tumor-suppressing activity. Elevated levels have recently been associated with stage elevation on hepatocellular carcinoma as well [46].

Hsa-miR-31 is predominantly reported as a pro-oncogenic molecule in the literature 30, [47–49]. Increased levels of hsa-mir-31 are associated with hepatocellular, breast, and colorectal carcinoma [50–52]. While miR-31-5p has been reported as upregulated in UC by Juracek et al. and identified as a target of KRT6 by Chen et al. [25], in our study, there was no significant difference in the expression of miR-31-5p between bladder cancer patients and the control group. However, significantly elevated levels of this miRNA were observed in basal-like tumors, as opposed to luminal-like tumors. This finding is consistent with the association previously proposed by Chen et al. [25]. Although levels of this miRNA showed a marginally significant increase in patients with CIS and correlated with larger tumor size, its role was complementary.

## Conclusion

In this exploratory, single-center study, urinary cell-free miR-93-5p and miR-191-5p showed potential as non-invasive biomarkers associated with basal-like and luminal-like molecular subtype characteristics of invasive urothelial carcinoma. miR-93-5p was associated with basal-like tumor features linked to aggressive behavior, whereas reduced miR-191-5p expression was associated with the presence of invasive urothelial carcinoma. miR-31-5p appears to play a complementary role, particularly in the context of carcinoma in situ. Although these findings suggest possible clinical applicability, external validation in larger, independent cohorts is required before routine clinical implementation.

## Limitations

This study has several limitations that should be considered when interpreting the findings. First, the cohort size was modest (*n* = 92), particularly after stratification into molecular subgroups, which limits statistical power and may increase the risk of effect-size inflation in subgroups and ROC-based analyses. Secondly, molecular subtyping relied on quantitative IHC rather than comprehensive transcriptomic or genomic (omics) profiling. While IHC-based classification using established markers provides a practical and clinically accessible surrogate aligned with the -omics classifications system, it may not capture the full molecular complexity revealed by RNA sequencing or microarray approaches. Third, the analyses presented are primarily univariable in nature. Given the limited sample size, multivariable modeling, adjusting for potential confounders such as age, tumor stage, grade, and carcinoma in situ, was not performed to avoid model overfitting and unstable estimates. Accordingly, all diagnostic performance metrics should be interpreted as exploratory, and external validation in larger, independent cohorts is required to confirm these observations.

## Supplementary Information

Supplementary Material 1.

Supplementary Material 2.

## Data Availability

The clinical patient data supporting the findings of this study are retained within the Urology Department at Cerrahpasa Medical Faculty and are not publicly available due to their containing information that could compromise patient privacy. Access is restricted and requires a direct formal requisition to the corresponding author.

## Abbreviations

AUC: Area Under the Curve
cDNA: Complementary DNA
CI: Confidence Interval
CIS: Carcinoma In Situ
Ct: Threshold Cycle
EMT: Epithelial-Mesenchymal Transition
IHC: Immunohistochemistry
IQR: Interquartile Range
LVI: Lymphovascular Invasion
MIBC: Muscle-Invasive Bladder Cancer
miRNA: microRNA
mRNA: Messenger RNA
NGS: Next-Generation Sequencing
NMIBC: Non-Muscle Invasive Bladder Cancer
RB: Retinoblastoma Protein
RISC: RNA-Induced Silencing Complex
ROC: Receiver Operating Characteristic
qRT-PCR: Quantitative real-time PCR
SD: Standard Deviation
SPSS: Statistical Package for the Social Sciences
TCGA: The Cancer Genome Atlas
TNM: Tumor, Node, Metastasis
TURBT: Transurethral Resection of Bladder Tumor
UC: Urothelial Carcinoma
UNG: Uracil-N-Glycosylase
