# Supplementary figures and images for: Clinical utility of cell-free urine miR-93-5p, miR-191-5p, miR-31-5p for invasive urothelial carcinoma detection and immune signature-based subtyping
Source: BMC Urol. 2026 Jan 15;26:41. doi: 10.1186/s12894-026-02047-y (PMC12892541; doi:10.1186/s12894-026-02047-y)

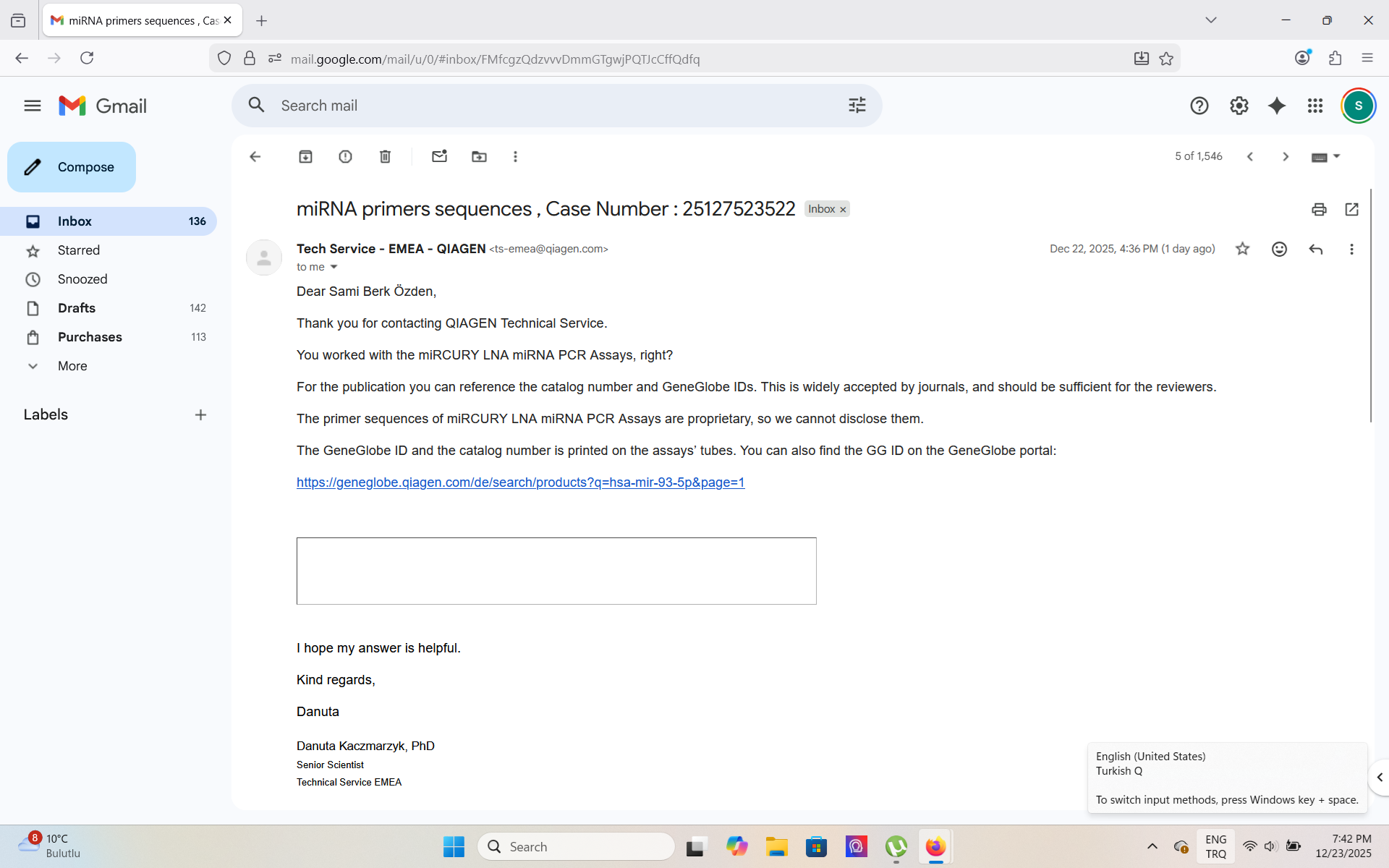

Supplement: Supplementary file 1 — Supplementary Material 1. [file 12894_2026_2047_MOESM1_ESM.png]
