# Supplementary material for: Clinical utility of cell-free urine miR-93-5p, miR-191-5p, miR-31-5p for invasive urothelial carcinoma detection and immune signature-based subtyping
Source: BMC Urol. 2026 Jan 15;26:41. doi: 10.1186/s12894-026-02047-y (PMC12892541; doi:10.1186/s12894-026-02047-y)
Supplement: Supplementary file 2 — Supplementary Material 2. [file 12894_2026_2047_MOESM2_ESM.pdf]

LABGEN LABORATUVAR SİSTEMLERİ SAN.TİC.AŞ
MİTHATPAŞA CAD.NO:79 K.1 D.1BALÇOVA
35330 IZMIR / IZMIR
Tel: 0232 2780677
Fax: 0232 2780699
e-Posta: info@labgen.com.tr
Vergi Dairesi: BALÇOVA
VKN: 6070383376
Ticaret Sicil No:
Mersis No: 0607038337600001
Firma Tanımlayıcı No: 2667269065354
İşletme Merkezi: IZMIR

labgen
Laboratuvar Sistemleri San.Ve Tic.A.Ş.

e-İRSALİYE

**Özelleştirme No**	TR1.2
**Senaryo**	TEMELIRSALIYE
**İrsaliye Tipi**	SEVK
**İrsaliye No**	LIA2022000002150
**İrsaliye Tarihi**	06-12-2022
**İrsaliye Zamanı**	14:59:17
**Sevk Tarihi**	06-12-2022
**Sevk Zamanı**	15:12:16
**CEOMED Belge No**	LI006601

SAYIN
İSTANBUL ÜNV.CERRAHPAŞA STRATEJI GELİŞTİRME DAİRE BAŞKANLIĞI

34320 ISTANBUL/ ISTANBUL
Vergi Dairesi: HOCAPAŞA
VKN: 4690969823

SEVK ADRESİ
İSTANBUL ÜNV.CERRAHPAŞA STRATEJI GELİŞTİRME DAİRE BAŞKANLIĞI
İDARİ BİRİMLER BİNASI AVCILAR YERLEŞKESİ HOCAPAŞA V.D./VKN: 4690970116 (AVCILAR VERGİ DAİRESİ)
ISTANBUL / ISTANBUL
ETTN: D512ABC8-8327-4F88-BE4C-EF21E371A4BB

Sıra No	Stok Kodu	UTS Kodu	Stok Adı	Lot	Miat	Ana Birim	Miktar
1	339306	KAPSAMDIŞI	hsa-miR-191-5p miRCURY LNA miRNA PCR Assay (200)	YP00204306		,01 KUTU	1,0 ADET
2	339306	KAPSAMDIŞI	hsa-miR-31-5p miRCURY LNA miRNA PCR Assay (200)	YP00204715		,01 KUTU	1,0 ADET
3	339306	KAPSAMDIŞI	hsa-miR-93-5p miRCURY LNA miRNA PCR Assay (200)	YP00204236		,01 KUTU	1,0 ADET
4	339340	KAPSAMDIŞI	miRCURY LNA RT Kit (8-64 rxn)	77202643	06/08/2023	1 KUTU	1,0 ADET
5	339345	KAPSAMDIŞI	miRCURY LNA SYBR Green PCR Kit (200)	77202468	21/09/2023	,01 KUTU	1,0 ADET
6	217184	KAPSAMDIŞI	miRNeasy Serum/Plasma Kit (50)	172036087		1 KUTU	1,0 ADET

Açıklamalar

Not: İSTANBUL ÜNV.CERRAHPAŞA BAP KOOR.BR.
Not: VD.AVCILAR 2.V.N: 4690970116
Not: PROJE KODU: TTU-2022-36413
Not: PROJE YÜRÜTÜCÜSÜ: DOÇ.DR.ÇETİN DEMİRDAĞ
Not: Gönderim Şekli:Kağıt
Not: Kurum Adı :İSTANBUL ÜNV.CERRAHPAŞA STRATEJİGELİŞTİRME DAİRE BAŞKANLIĞI
Not: İşin Adı :MESANE KANSERİ OLAN TURT YAPILAN VAKALARDA İLK İDRAR VE MESANE YI
Not: İşlem Tipi :02 FATURALI SEVK

Taşıyıcı Bilgileri

Taşıyıcı Firma: VKN: 6070383376, LABGEN A.Ş./İSTANBUL ŞUBE
Araç plaka numarası: 35 BGF 006
Şoför: ONUR KARA , TCKN: 35314364470

Teslim Eden	Teslim Alan
